# U.S. POINTER: Study Design, Recruitment, and Baseline Participant Characteristics of a Large Multisite Multidomain Lifestyle Intervention Trial

**DOI:** 10.1002/alz70860_096522

**Published:** 2025-12-23

**Authors:** Rachel A. Whitmer, Heather M Snyder, Marcus Hill, Chartay Robinson, Ashley Alexander, Tara Tang, Oanh L. Meyer, Elizabeth Omotoye, Kelvin Williams, Laura Lovato, Nancy Woolard, Miia Kivipelto, Mark A. Espeland, Laura D Baker, Maria C. Carrillo, U. S. POINTER Study Group

**Affiliations:** ^1^ University of California, Davis, Davis, CA, USA; ^2^ Alzheimer's Association, Chicago, IL, USA; ^3^ Wake Forest University School of Medicine, Winston Salem, NC, USA; ^4^ AARP Northern Illinois, Chicago, IL, USA; ^5^ Kelsey Research Foundation, Houston, TX, USA; ^6^ Butler Hospital, Providence, RI, USA; ^7^ University of California, Davis School of Medicine, Sacramento, CA, USA; ^8^ Advocate Aurora Health, Chicago, IL, USA; ^9^ Wake Forest University, Winston Salem, NC, USA; ^10^ Wake Forest University School of Medicine, Winston‐Salem, NC, USA; ^11^ Division of Clinical Geriatrics, Center for Alzheimer Research, Department of Neurobiology, Care Sciences and Society, Karolinska Institutet, Stockholm, Sweden; ^12^ FINGERS Brain Health Institute, Stockholm, Sweden; ^13^ Wake Forest University, Winston‐Salem, NC, USA; ^14^ Medical & Scientific Relations Division, Alzheimer's Association, Chicago, IL, USA

## Abstract

**Background:**

The U.S. Study to Protect Brain Health through Lifestyle Intervention to Reduce Risk (U.S. POINTER) is a Phase 3, multicenter, 2‐year randomized controlled trial assessing two multidomain lifestyle interventions in older adults at risk for cognitive decline. Key goals were to recruit a large, diverse, and representative cohort of older adults at risk for cognitive decline and dementia and to test whether 2 years of multidomain lifestyle modification could protect cognitive function.

**Methods:**

Recruitment efforts engaged nearly 17,000 individuals using electronic health records and grassroots strategies, leveraging outreach initiatives and trusted community partnerships in five U.S. regions. The enrollment goal was 2,000 participants, with at least 23% from underrepresented racial and ethnic groups, 50% women, and diversity in education and socioeconomic status. Both interventions targeted physical activity, nutrition, cognitive/social challenge and cardiometabolic risk reduction, but differed in intensity and accountability. The primary outcome, a global cognitive composite score, was assessed at baseline and every six months.

**Results:**

A total of 2,111 participants (aged 60‐79 years) were enrolled, with 69% women, 31% from underrepresented ethnoracial groups, 18% from neighborhoods with moderate or high socioeconomic deprivation, and 30% without a college degree. Participants were cognitively unimpaired at study entry but at risk due to sedentary behavior, poor diet, family history of memory impairment, and suboptimal cardiometabolic health. At baseline, participants had mild elevated blood pressure (mean SBP=131mmHg), prediabetes (mean fasting glucose=102mg/dl, HbA1c=5.9%), and mild dyslipidemia (total cholesterol=194mg/dl; LDL‐C=112mg/dl). They reported following suboptimal diets (mean MIND diet score=7.0/14) and had room for improvement on a test of global cognition (mean TICSm=39/50) and physical function (mean SPPB=10.9/12). Seventy‐eight percent reported first‐degree family history of memory loss, and 30% were APOE‐e4 carriers (2.9% homozygotes). Participation rate in at least one of four ancillary studies was 73%, which was uniform across sociodemographic groups.

**Conclusion:**

The cohort meets study goals for diversity and risk, providing a strong foundation for assessing multidomain lifestyle intervention effects on cognitive function and other outcomes that will generalize to large portions of the U.S. population at risk for cognitive decline and dementia.

